# Biomechanical comparison of inverted triangle and L-shaped screw configurations with medial buttress and anteromedial support plate in Pauwels type III femoral neck fractures

**DOI:** 10.1007/s00402-026-06352-x

**Published:** 2026-06-06

**Authors:** Murat Erem, Utku Demirtaş, Savaş Yıldırım, Eşref Selçuk, Cem Çopuroğlu

**Affiliations:** 1 Department of Orthopaedics and Traumatology, Trakya University, Edirne, Turkey; 2 Department of Orthopaedics and Traumatology, Sultan 1.Murat State Hospital, Edirne, Turkey

**Keywords:** Femoral neck fracture, Pauwels type III, Biomechanical testing, Medial buttress plate, Anteromedial support plate, Internal fixation

## Abstract

**Introduction:**

Surgical stabilization of Pauwels Type III femoral neck fractures remains a significant challenge due to high vertical shear forces. While the medial buttress plate is a recognized solution, it requires extensive deep dissection. This study aims to compare the biomechanical performance of various screw configurations combined with either a medial buttress or anteromedial support plate.

**Materials and methods:**

Twenty-five third-generation synthetic femurs were used to create a standardized 70-degree (Pauwels III) fracture model. Specimens were divided into five groups (*n* = 5): (A) Inverted triangle (IT) screws with a Pauwels screw, (B) Inverted triangle (IT) with medial buttress plate (MBP), (C) Inverted triangle with anteromedial support plate (ASP), (D) L-configuration with medial buttress plate (MBP), and (E) L-configuration with anteromedial support plate (ASP). Axial loading was applied at 2 mm/min until construct failure, defined objectively by the real-time force-distance curve.

**Results:**

Although no statistically significant difference was found between groups (*p* = 0.102), a large effect size was observed (η² = 0.309). Group C (IT + ASP) demonstrated the highest mean failure load (1695 ± 494.6 N). Conversely, Group E (L-configuration + ASP) exhibited the lowest stability (977.2 ± 195.4 N) with a remarkably narrow standard deviation. The majority of failures occurred as transverse subtrochanteric fractures distal to the implants.

**Conclusion:**

The combination of an inverted triangle screw arrangement with an anteromedial support plate demonstrated comparable biomechanical stability to the medial buttress plate, while offering a potentially safer surgical corridor. Conversely, pairing L-shaped screw configurations with anteromedial support plates resulted in the lowest mean ultimate load-to-failure among the tested constructs, likely due to potential stress riser effects.

## Introduction

Femoral neck fractures in young adults are typically the result of high-energy trauma and are still considered a challenging problem to resolve [1, 2]. The objective of treatment in this patient group is to achieve anatomical reduction and stable fixation while preserving the biological viability of the femoral head [3]. However, in Pauwels Type III fractures, where the angle between the fracture line and the vertical plane exceeds 50 degrees, elevated shearing forces exerted on the fracture line and varus instability escalate the risk of severe complications, including fixation failure, nonunion (10–30%), and avascular necrosis (AVN) [1, 2, 4]. Conventionally, multi-cannulated screw fixation has been a prevalent treatment modality for such fractures. According to the results of biomechanical studies, the configuration of screws is found to be a critical factor in ensuring stability [5–7]. Although the classic inverted triangle (IT) configuration is the most commonly preferred method, modified screw arrangements with or without medial plates or L-configurations with medial plates may offer biomechanical advantages by providing a wider support surface [5, 6, 8]. A growing consensus exists that isolated screw fixation (regardless of configuration) may be inadequate under axial loads and may fail to prevent varus collapse in high-angle Pauwels III fractures [6, 9, 10]. To address this mechanical inadequacy, the concept of a medial buttress plate (MBP) has been developed. As demonstrated, the application of an MBP to the medial cortex has been shown to neutralize shearing forces, thereby enhancing the efficacy of screw fixation [11, 12]. In a similar vein, that plate support significantly reduced interfragmentary movement and angular deformity [13]. Although recent clinical studies suggest that MBP does not significantly increase the rate of AVN, the procedure is associated with a longer operation time and greater intraoperative blood loss compared with cannulated screws alone, supporting the assertion of increased procedural complexity [14]. This anatomical complexity poses a technical challenge and steeper learning curve compared to other approaches, necessitating precise execution to avoid iatrogenic vascular injury. Alternatively, Zhuang et al. [15] utilized an anteromedial support plate (ASP) technique via a modified anterior approach, primarily for irreducible fractures requiring open reduction. Although their primary indication was reducibility, this approach inherently avoids the deep medial dissection required for buttress plating, offering a potentially safer corridor with reported low complication rates. However, a review of the extant literature reveals a lack of studies that directly compare the biomechanical effectiveness of the ASP with that of the MBP. Furthermore, the literature does not thoroughly explore how the combination of these plates with different screw configurations (inverted triangle vs. L-shaped) affects stability. Therefore, this study aimed to compare the biomechanical performance of inverted triangle versus L-shaped screw configurations combined with MBP or ASP support in a standardized Pauwels Type III femoral neck fracture model. The hypothesis of this study was that biomechanical stability is highly dependent on the specific combination of screw and plate position and that the ASP would provide a biomechanically comparable alternative to the MBP when combined with an appropriate screw configuration, outperforming standard non-plate augmentation.

## Materials and methods

### Study design overview

This study was designed as a controlled, experimental in vitro biomechanical investigation comparing five different fixation constructs for Pauwels type III femoral neck fractures. A total of twenty-five third-generation composite femurs (Selbones Research Lab.,, Kayseri, Türkiye), with a uniform Caput-Collum-Diaphyseal (CCD) angle of 130°, were utilized for all experimental groups to eliminate morphological variations designed to replicate the mechanical properties of human cortical and cancellous bone. The use of composite femurs provided uniform material properties and minimized inter-specimen variability commonly encountered with cadaveric specimens [16].

### Specimen preparation and mounting

**Fig. 1:**
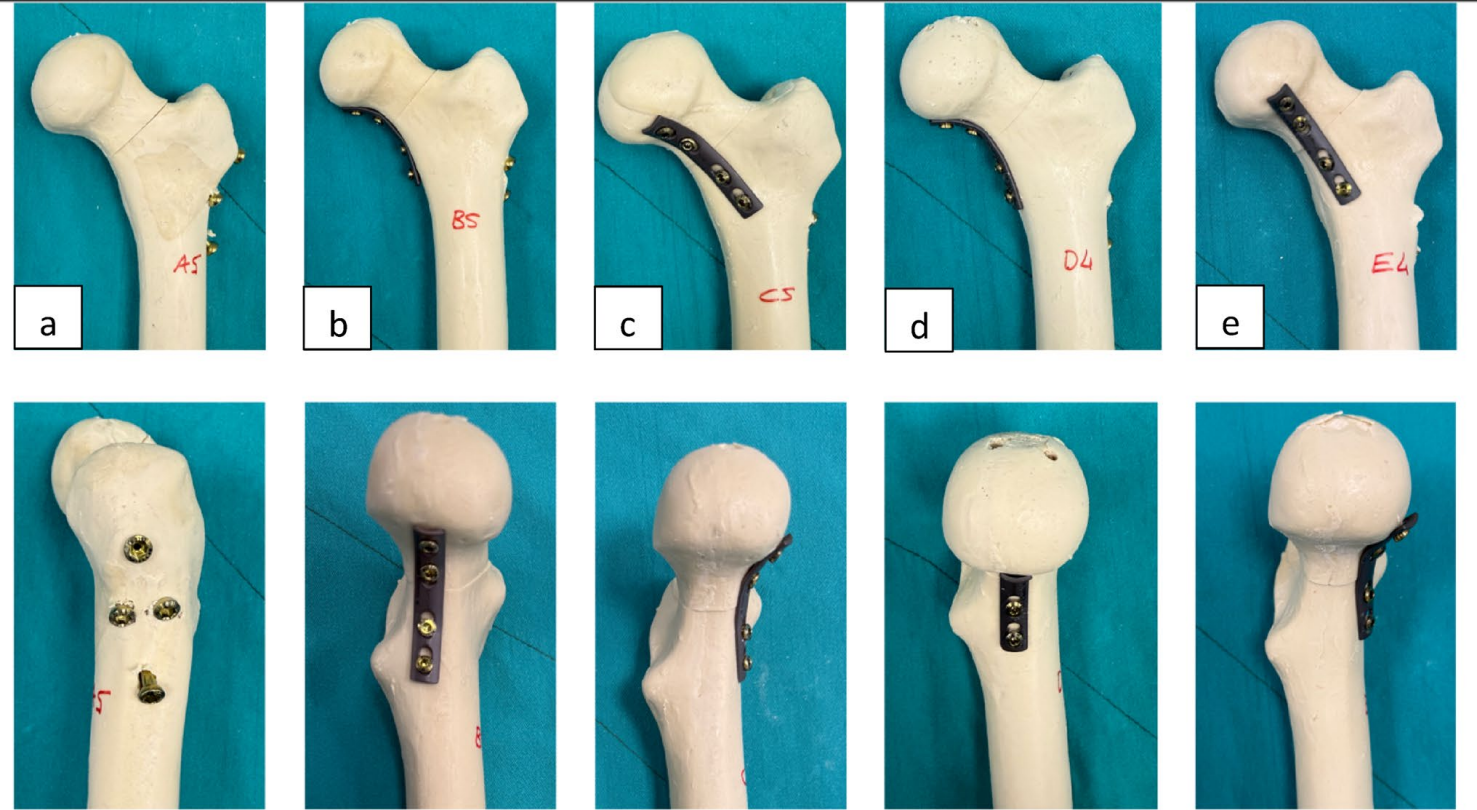
Anterior (top row) and lateral (bottom row) macroscopic views of the fixation constructs: **a** Inverted Triangle with Pauwels screw, **b** Medial Buttress Plate with Inverted Triangle, **c** Anteromedial Support Plate with Inverted Triangle, **d** Medial Buttress Plate with L-Configuration, and **e** Anteromedial Support Plate with L-Configuration

To allow secure fixation within the testing apparatus, each femur was transected transversely at the proximal diaphyseal level. A custom-designed metallic fixation jig was manufactured to standardize specimen positioning during mechanical testing. Specimens were mounted with a 7° inclination relative to the loading axis to simulate physiological axial loading conditions (Fig. 1).

### Fracture model creation

To simulate high-energy injuries in young patients, a standardized Pauwels Type III fracture line was created in all specimens; an oscillating saw was used to create a precision osteotomy passing through the middle third of the femoral neck region and forming a 70-degree angle with the horizontal plane.

### Fixation groups, screw configuration, and standardization

Specimens were randomly allocated into five groups (*n* = 5 per group) (Table 1). In all groups, 6.5 mm partially threaded cannulated screws were used. Group A was established as the clinical baseline reference construct, representing a standard, widely utilized non-plate fixation strategy for Pauwels Type III fractures [9, 17]. Inverted Triangle Configuration (Groups A, B, C): Three screws were placed in a parallel inverted triangular pattern, with one screw inferiorly along the calcar and two screws superiorly (anterior and posterior). L-Configuration (Groups D, E): Three screws were placed in an L-shaped pattern, with one screw inferiorly along the calcar and two screws vertically aligned along the anterior cortex (Fig. 1).

All constructs utilized three 6.5-mm cannulated screws with a 16-mm thread length. For the L-configuration, two 100-mm screws and one 90-mm screw were used. The inverted triangle configuration consisted of two 90-mm screws and one 100-mm screw. In Group A, the additional Pauwels screw measured 85 mm in length. These specific screw lengths were selected based on their respective trajectories to ensure a uniform subchondral depth across all constructs.

**Table 1:** Description of fixation constructs used in the study

Group	Screw configuration	Additional fixation	Plate position
A	Inverted triangle	Pauwels screw	–
B	Inverted triangle	Medial buttress plate	Medial (6 o’clock)
C	Inverted triangle	Anteromedial support plate	Anteromedial (4 o’clock)
D	L-configuration	Medial buttress plate	Medial (6 o’clock)
E	L-configuration	Anteromedial support plate	Anteromedial (4 o’clock)

**Fig. 2:**
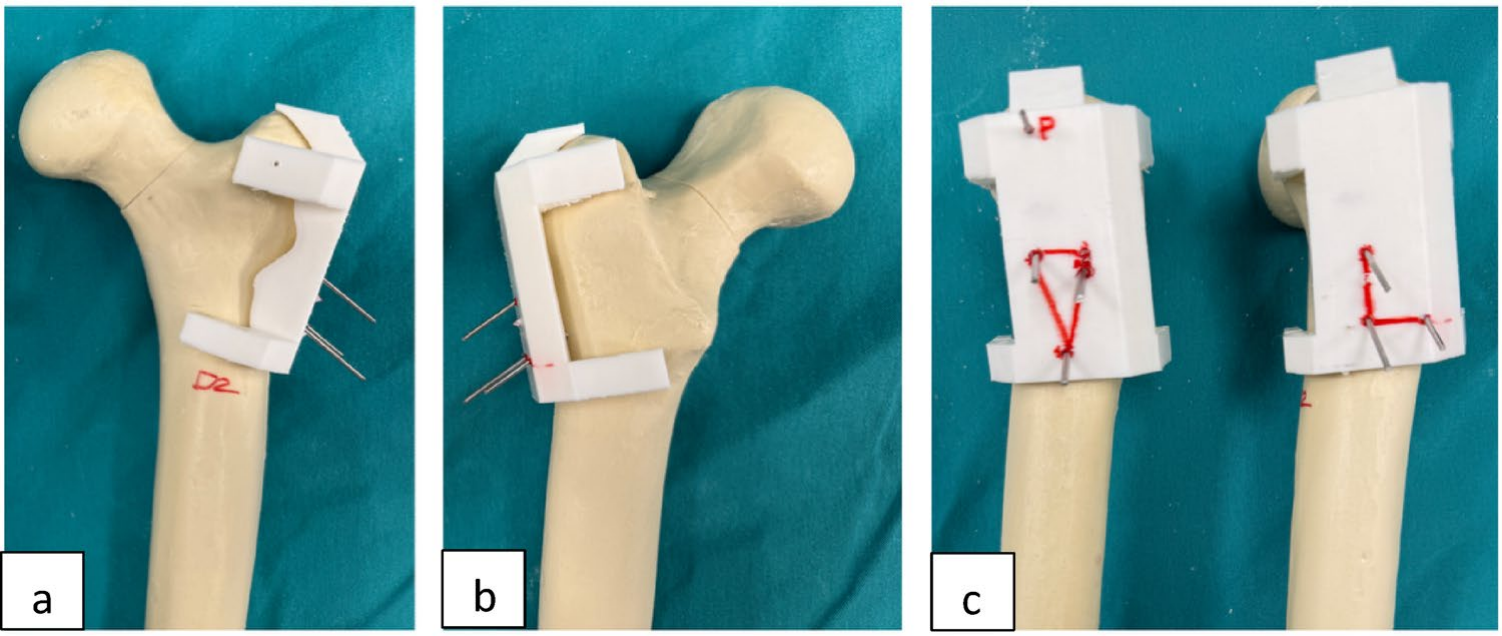
Specimen-specific 3D-printed drill guide for trajectory standardization: **a** Anterior, **b** Posterior, and **c** The lateral views demonstrate the entry points for inverted triangle and L-configuration patterns, with the ‘P’ marker indicating the standardized entry point for the Pauwels screw

To ensure precise and reproducible cannulated screw placement, each composite femur was three-dimensionally scanned, and a specimen-specific drill guide was designed using CAD software (Shapr3D, Ireland). The guides were subsequently manufactured using a 3D printer based on Fused Deposition Modeling (FDM) technology with Polylactic Acid (PLA) filament. These guides enabled standardized screw trajectories for both inverted triangle and L configurations, minimizing operator-dependent variability (Fig. 2).

## Plate fixation

**Fig. 3:**
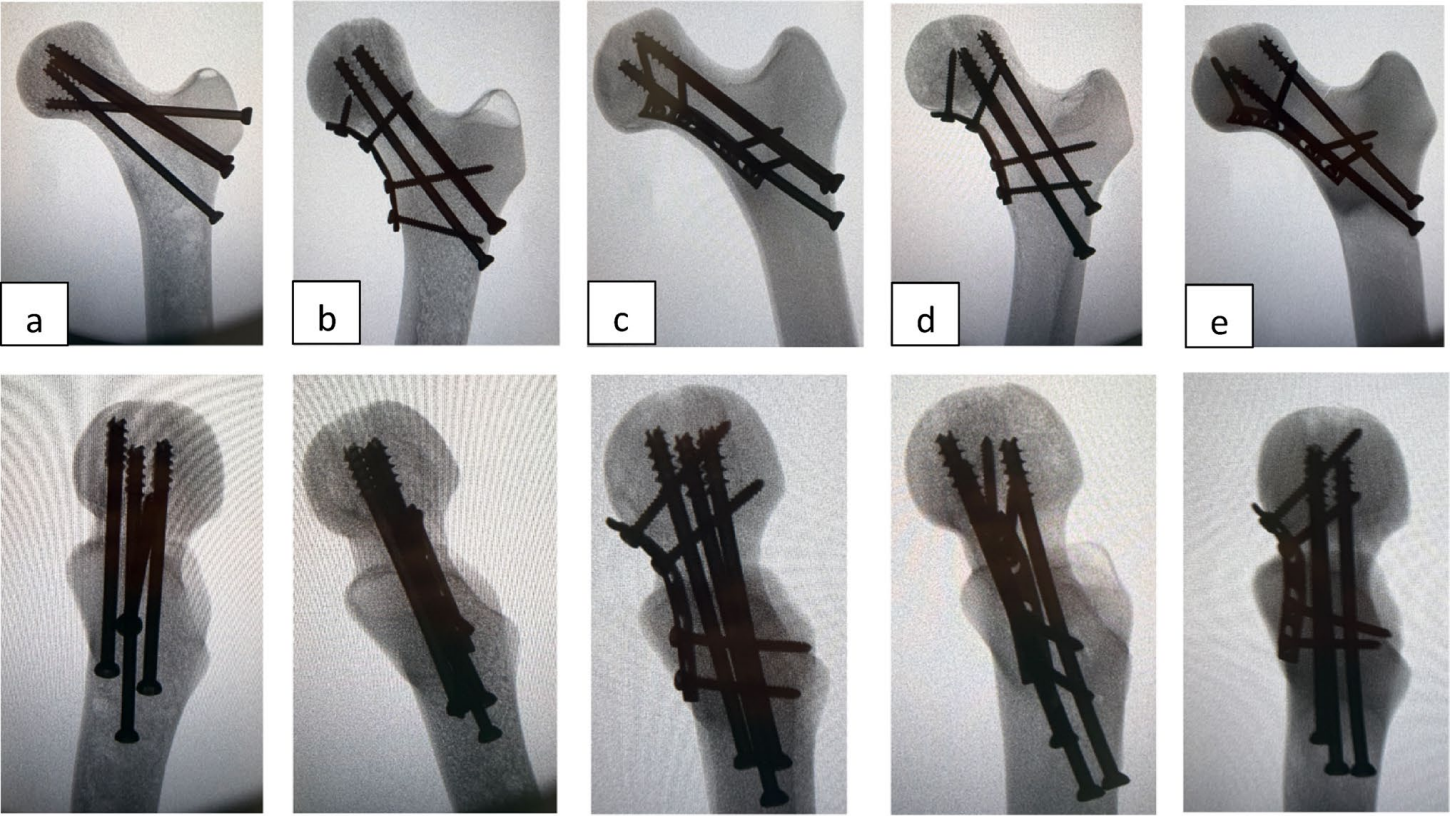
Anteroposterior (top row) and lateral (bottom row) radiographs of the fixation constructs: **a** Inverted Triangle with Pauwels screw, **b** Medial Buttress Plate with Inverted Triangle, **c** Anteromedial Support Plate with Inverted Triangle, **d** Medial Buttress Plate with L-Configuration, and **e** Anteromedial Support Plate with L-Configuration

A 4-hole dynamic compression plate (DCP) was used for all plated constructs. The plate’s dimensions were 3.5 mm thickness, 10 mm width, and 50 mm length. All implants were manufactured from medical-grade titanium alloy (Mikron^®^, Istanbul, Türkiye). Plate position relative to the femoral neck was described using a clock-face system: 12:00 cephalad, 3:00 anterior, 6:00 caudad, and 9:00 posterior. Medial buttress plates were positioned at the 6 o’clock orientation, while anteromedial support plates were positioned at the 4 o’clock orientation with an inclination of approximately 45° relative to the femoral neck. All plates were fixed using non-locking cortical screws (3.5 mm diameter, titanium). In Groups C and E, proximal screw lengths were 40 mm and 36 mm, and distal screw lengths were 40 mm and 46 mm. In Groups B and D, proximal screw lengths were 30 mm, and distal screw lengths were 40 mm and 42 mm (Fig. 3).

### Mechanical testing protocol

**Fig. 4:**
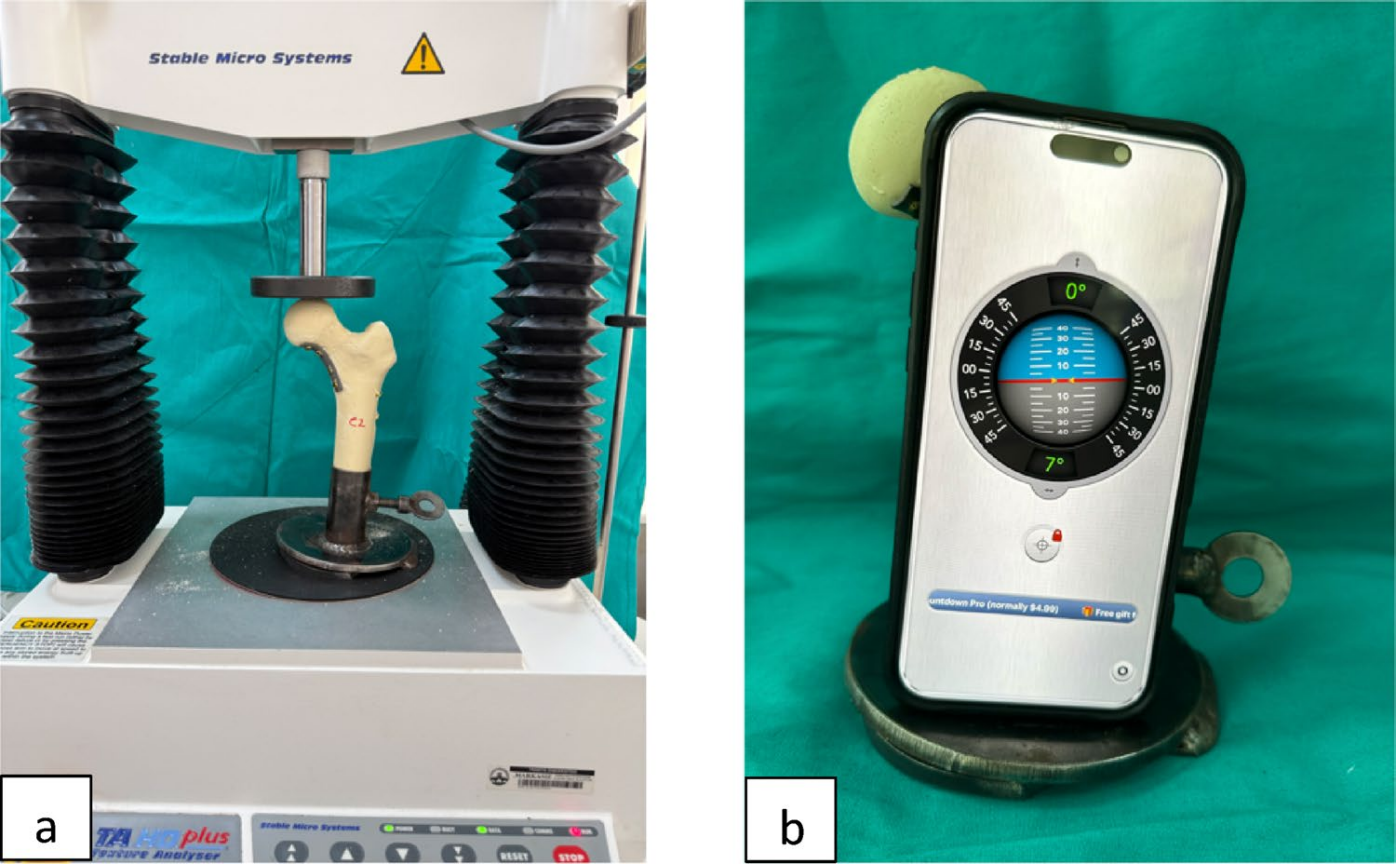
Experimental setup used for axial biomechanical testing. **a** The mechanical testing device demonstrating axial loading of the specimen, including the custom fixation apparatus securing the femoral shaft. **b** Verification of the 7° inclination angle used to simulate physiological axial loading conditions, confirmed using a digital inclinometer application

Specimens were mounted on a mechanical testing device with a 7° adduction in the frontal plane and aligned vertically in the sagittal plane to replicate the single-legged mid-stance phase of gait [18, 19] (Fig. 4).

To determine the initial construct stiffness and ultimate maximum strength, a vertical load was applied to the superior aspect of the femoral head. Mechanical testing was performed using a TA. HD Plus Texture Analyzer (Stable Micro Systems, UK). The experimental setup applied a continuous, monotonic axial load to failure at a constant displacement rate of 2 mm/min until failure occurred [19, 20], which most commonly manifested as a transverse subtrochanteric fracture distal to the most inferior cannulated screw. To isolate the evaluation of absolute mechanical stability under single-load scenario, no preconditioning cyclic loading or fatigue protocols were applied at any stage of the testing.

### Outcome measures and failure definition

**Fig. 5:**
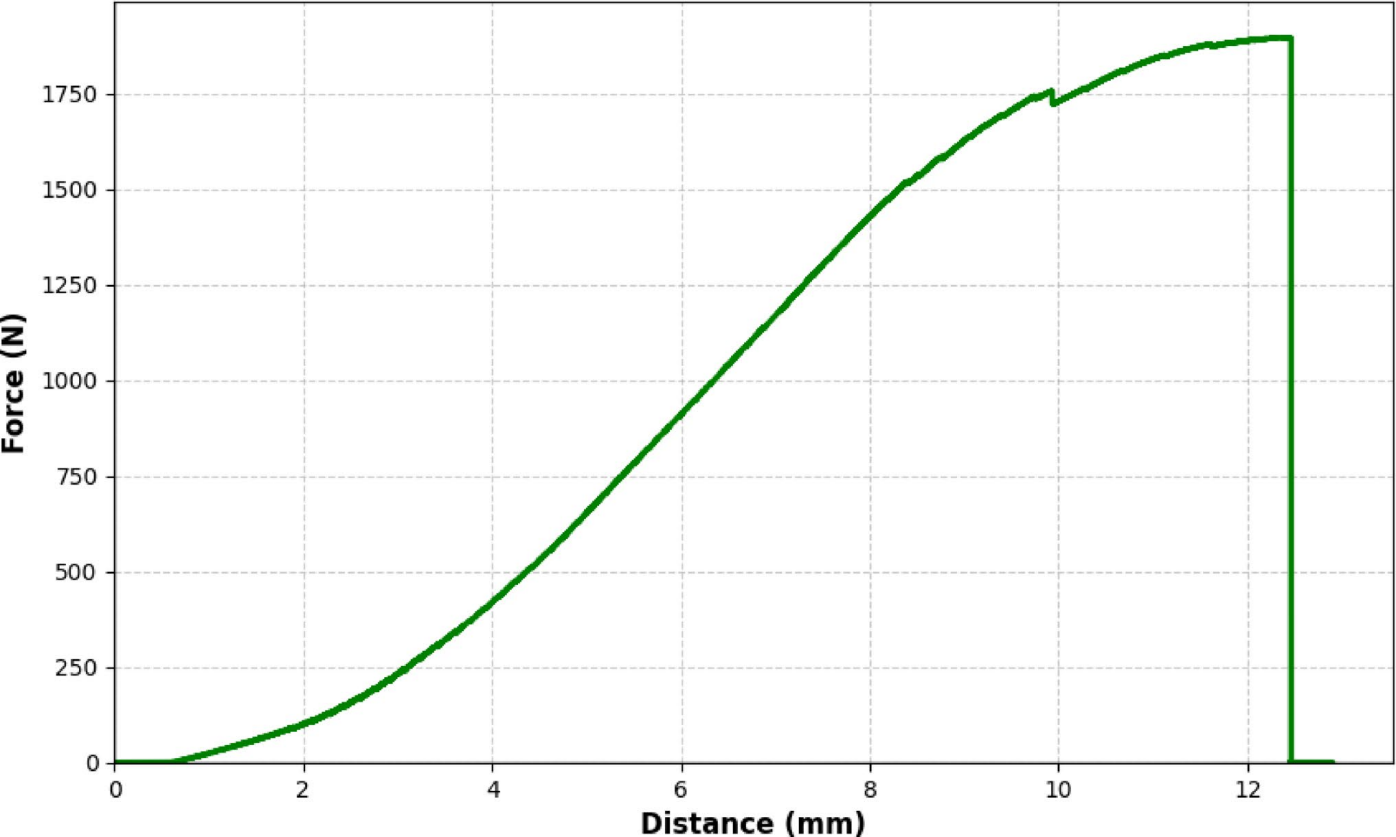
A real-time force-distance curve obtained during axial loading (Sample A1, inverted triangle configuration with Pauwels screw). The objective failure point is clearly identifiable as the peak force immediately preceding the sudden, sharp drop in the curve

For the purposes of outcome assessment, the primary endpoint was defined as construct failure. The exact failure load was objectively defined as the peak load immediately preceding the sudden, sharp drop on the real-time force-distance curve generated by the testing software (FarPoint Technologies) (Fig. 5). This objective mechanical failure point was found to consistently correlate with a 2-mm displacement threshold at the fracture site, which was verified via continuous visual monitoring of the fracture interface circumferentially (360°) as a qualitative confirmation.

### Statistical analysis

Mechanical testing data were collected using FarPoint Technologies software and analyzed using JASP statistical software (version 0.95.4, University of Amsterdam). Descriptive statistics were calculated for all groups. Normality was assessed using the Shapiro–Wilk test. Homogeneity of variances was evaluated using Levene’s test. As parametric assumptions were met, one-way analysis of variance (ANOVA) was performed to compare groups, followed by Tukey post-hoc tests. Effect sizes, standard deviations (SD), and 95% confidence intervals (CI) were calculated. Statistical significance was set at *p* < 0.05. Data visualization and the generation of figures were performed using custom scripts written in the Python programming language (utilizing the Matplotlib library) to ensure high-resolution and precise data representation.

## Results

### Mechanical testing outcomes

**Fig. 6:**
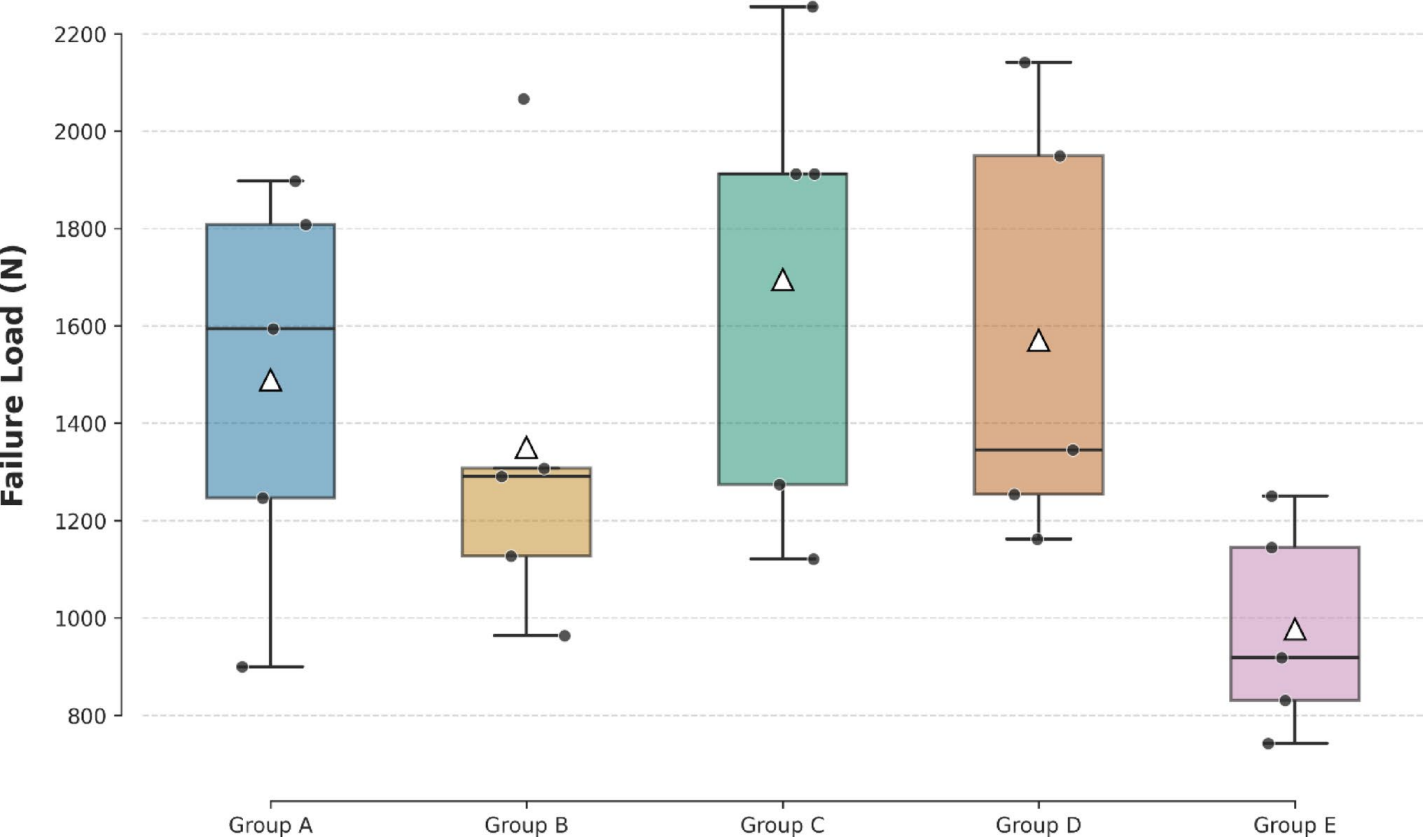
The failure load (newton) for five fixation methods. The horizontal line inside each box indicates the median, while the white triangle (△) represents the mean value. Black dots represent individual sample data points

All specimens successfully underwent axial biomechanical testing until failure. No technical issues were encountered during specimen preparation, fixation, or mechanical loading. The mean axial load to failure for each fixation group is summarized in Table 2, and the comparative distribution of failure loads is illustrated in Fig. 6.

**Table 2:** Descriptive statistics of axial load to failure for all fixation constructs

Group	Mean± SD (*N*)	Median (*N*)	Min (*N*)	Max (*N*)	CV (%)	95% CI^a^
Group A	1489±400.6	1594	899.3	1897	2.6%	991.5–1986
Group B	1351±423.1	1291	1504.6	1880.6	3.1%	825.2–1876
Group C	1695±494.6	1912	1527.8	2403.4	2.9%	296.4–1421
Group D	1570±477.3	1345	1162	2141	3%	977.4–2163
Group E	977.2±195.4	918	742.4	1251	2%	734.5–1220

^a^95% CI mean lower and upper bound

*N* newton, *SD* standard deviation, *Min* minimum, *Max* maximum, *CV* coefficient of variation, *CI* confidence interval

In the statistical comparison between the five groups, the Levene test revealed that the variance distribution of the groups was homogeneous (*p* = 0.173). One-way ANOVA revealed no statistically significant difference was found between the groups in terms of axial load to failure values (F(4,20) = 2.232, *p* = 0.102). However, the observed effect size was large (η² = 0.309), indicating that 31% of the variance in failure load was attributable to fixation method. Post-hoc Tukey analysis did not demonstrate any significant pairwise differences between groups (*p* > 0.05). The mean ± SD axial load to failure was Group A: 1489**±** 400.6 N, Group B: 1351**±** 423.1 N, Group C: 1695**±** 494.6 N, Group D: 1570**±** 477.3 N, Group E: 977.2**±** 195.4 N. In Group C, the highest mean fail-to-load resistance was observed; Groups B and D exhibited similar resistance profiles, and Group E showed the lowest resistance. While Group C and Group E showed the greatest difference, it was not statistically significant (*p* = 0.098).

### Effect size and variability

The coefficients of variation (CV) were similar among groups, suggesting consistent mechanical behavior and minimal inter-specimen variability across fixation constructs (Table 2).

Despite the absence of statistically significant differences, effect size analysis revealed a large effect for the fixation construct on axial load to failure (η² = 0.309), indicating that a substantial proportion of the observed variance was associated with the fixation method. CI showed considerable overlap among groups, supporting a comparable mechanical performance across constructs (Table 2).

### Failure modes

Failure mode analysis revealed a consistent fracture pattern across all groups. The majority of specimens failed via a transverse subtrochanteric fracture distal to the most inferior cannulated screw. No cases of implant breakage, screw pull-out, or plate deformation were observed.

## Discussions

A notable finding was that the IT screw configuration with ASP (Group C) demonstrated the highest mean failure load (1695 ± 494.6 N) among all tested constructs, whereas the L-configuration with ASP (Group E) exhibited the lowest mean failure load (977.2 ± 195.4 N). Despite the absence of a statistically significant difference (*p* = 0.102) between the groups, the large effect size suggests that the specific spatial arrangement of the cannulated screws is a critical determinant of overall construct stability when utilizing ASP. Consequently, the choice of screw configuration should be carefully considered during preoperative planning and clinical decision-making.

While the MBP is often considered for neutralizing vertical shear forces, the constructs of the ASP (Group C) demonstrated a higher mean failure load compared to the MBP groups (Groups B and D). This suggests that the ASP provided additional resistance against the anterior component of the shear displacement. Although our study protocol focused on axial loading, the high mean failure load observed in Group C aligns with the findings of Fan et al. [19], who reported that ASP significantly enhances torsional strength and torsional stiffness. This dual-function support mechanism may explain why Group C demonstrated a higher mean axial load-to-failure than both MBP groups despite the advantages of medial buttressing described in previous studies [12, 14, 21, 22]. Zhuang et al. [15] demonstrated in a clinical series that the anteromedial approach is a safe alternative in cases where closed reduction is not possible and that the plate applied from this region stabilizes the fracture line. Our biomechanical findings support this clinical observation by demonstrating that the plate applied from this region avoids the technical complexity and deep dissection inherent to medial buttress plating while providing a comparable load-bearing capacity. Although not statistically significant, the fact that Group C (1695 N) demonstrated a higher mean failure load than MBP constructs, regardless of whether they utilized an IT (Group B: 1351 N) or L configuration (Group D: 1570 N), suggests potential clinical relevance for the ASP.

The constructs utilizing MBP groups (Groups B and D) demonstrated consistent stability, with failure loads of 1351 N and 1570 N, respectively, and were consistent with the existing literature [19, 21]. Previous studies have established that the MBP is used in Pauwels type III fractures by neutralizing shear forces in the inferior cortex of the femoral neck [12, 14, 21, 22] and the buttress effect provided by the MBP is superior to isolated screw fixation in preventing varus collapse [11, 12]. Similarly, in a cadaver study, Nwankwo et al. [13] showed that sliding hip screws with MBP significantly reduced both shearing and angular displacement in comparison to derotational screws. However, from a surgical practice perspective, the deep dissection required to access the medial region carries the risk of injury to the medial circumflex femoral artery. Our experimental data aligns with these models, suggesting that comparable mechanical benefits can be achieved via the ASP, which offers a less complex surgical approach.

The most salient, paradoxical finding of our study, which appears to contradict the extant literature, is that the L-shaped screw configuration combined with the ASP (Group E) exhibited the lowest mean failure load (977.2 ± 195.4 N) and it also exhibited low variance. The fact that the standard deviation is so narrow indicates that the failure in this configuration is not random; rather, it points to a consistent structural weakness. However, L-shaped or off-axis screw arrangements provide a larger surface area, thereby distributing stress and offering superior performance, particularly under torsional loads [6, 8]. The failure of this group in our study underscores the finding that increasing the number of implants does not guarantee increased stability. The proximity of the entry points of the L-configuration screws placed inferiorly and the screws of the ASP may have created a stress concentration point, which could have resulted in excessive damage to the bone stock within the limited volume of the femoral neck. This implies that the implants may have induced a stress riser effect, resulting in iatrogenic fracture line weakness rather than enhancing bone strength. This outcome serves as a critical warning to surgeons, underscoring the necessity to consider the potential three-dimensional spatial conflict of screw trajectories when planning combined fixation methods.

The isolated IT configuration augmented with a Pauwels screw (Group A) demonstrated a robust ultimate load-to-failure (1489 ± 400.6 N), reaffirming its role as a reliable clinical baseline even in the absence of plate support. Despite the observation in meta-analysis that isolated screw fixation has high failure rates (nonunion, AVN), the results of this study indicate that in early-stage weight-bearing simulations, the two proximal screws of the IT may be mechanically sufficient to counterbalance the lever arm effect [21]. However, as emphasized, screws may tend to loosen over time during the biological healing process [20, 23]. Therefore, although Group A demonstrated robust stability under static loading in our study, the implications of cyclic loads and micromotion in clinical practice must be considered. While our monotonic testing did not demonstrate a statistically significant difference, plate augmentation is theoretically intended to provide an additional safety margin against progressive varus collapse under physiological conditions.

A critical methodological consideration in our study was the standardization of screw lengths. Although varying screw lengths (90 mm and 100 mm) were utilized depending on the specific configuration, this variation does not constitute an uncontrolled mechanical variable. Rather, it reflects a clinical requirement to eliminate the confounding variable of uneven subchondral thread engagement. In clinical practice, maintaining a safe and consistent screw-apex distance is vital; placing screws too close to the weight-bearing apex can damage the principal compressive trabeculae and lead to severe complications, including osteonecrosis and subchondral collapse [24]. By tailoring screw lengths to their respective trajectories, we ensured that all screw tips consistently achieved a standardized subchondral depth. This approach maximized equal subchondral purchase across all groups, thereby maintaining the biomechanical validity of the comparisons.

Regarding the failure patterns, a recognized biomechanical failure mode in composite bone models was rigidly potted at the diaphysis, creating a stress riser at the distal implant-bone interface, which has been widely documented in comparable biomechanical studies [5, 12, 20]. However, the absence of primary failure at the femoral neck fracture itself indicates that all tested constructs provided sufficient stability to successfully transfer the applied axial loads distally. Thus, while the ultimate catastrophic failure highlights a diaphyseal stress concentration, the recorded failure loads remain a valid comparative indicator of the overall structural integrity and load-sharing capacity of the proximal fixations.

### Clinical implications

The findings suggest that the IT configuration with the ASP is a biomechanically comparable alternative to the MBP, offering similar stability while avoiding the risks of deep medial dissection. Caution is warranted when integrating varied fixation principles; in particular, pairing L-configuration screws with ASP is inadvisable, as our data indicates it may act as a stress riser and compromise the overall structural integrity of the construct.

### Limitations

Several limitations of this study should be acknowledged. Although the third-generation synthetic bone models utilized in the experiments provided standardization and eliminated the variability in bone quality found in cadaver studies, they did not reflect the contribution of the soft tissue, such as the capsule and ligaments, to stability and the biological response during the bone healing process. A post-hoc power analysis based on the observed effect size demonstrated a statistical power of 0.65; the sample size paralleled prior biomechanical studies on femoral neck fractures with composite models [5, 6]. This relatively low power may have impeded statistical significance for minor differences, notably between Groups B and C. Therefore, we explicitly acknowledge that the lack of statistical significance in our study does not prove definitive mechanical equivalence. While the absence of significant differences should be evaluated alongside effect sizes and confidence intervals, rather than solely p-values. Given the consistent failure patterns and similar variability across groups, the findings suggest comparable trends in mechanical behavior among the tested constructs. Consequently, larger sample sizes are required to definitively establish equivalence and further clarify subtle differences.

The experimental setup simulated isolated monotonic physiological axial loading with a 7-degree adduction angle; however, Pauwels type III fractures are also exposed to significant torsional forces during ambulation. Although this method is highly reliable for determining baseline construct stiffness and ultimate load-to-failure of initial fixation in a simulated, single-load scenario, it does not fully simulate physiological fatigue, micromotion, or repetitive stresses experienced during normal postoperative gait. Therefore, the absence of cyclic testing to evaluate progressive construct fatigue is a limitation.

Future research should focus on validating the stress riser effect in Group E using finite element analysis to visualize stress concentration points at the screw-plate interface. Additionally, subsequent studies that incorporate torsional and cyclic loading protocols are essential to evaluate the long-term dynamic stability of these configurations.

## Conclusions

The research findings indicate that combining an IT screw arrangement with an ASP delivers mechanical stability comparable to MBP fixation for Pauwels Type III femoral neck fractures. This approach also presents a reduced surgical risk profile by avoiding the need for extensive medial tissue dissection. In contrast, pairing L-shaped screw placement with an ASP yielded the lowest ultimate load-to-failure, suggesting that mismatched screw orientations can undermine the overall stability of the fixation construct. Therefore, when developing fixation plans, surgeons should emphasize ensuring the spatial alignment and compatibility of implants rather than simply maximizing the number of implants used.

## Data Availability

No datasets were generated or analysed during the current study.
